# The Longitudinal Relationship Between Brain Morphology and Obsessive-Compulsive Symptoms in Children From the General Population

**DOI:** 10.1016/j.jaacop.2023.11.003

**Published:** 2023-12-25

**Authors:** Cees J. Weeland, Chris Vriend, Henning Tiemeier, Odile A. van den Heuvel, Tonya White

**Affiliations:** a Amsterdam UMC location Vrije Universiteit Amsterdam, Amsterdam, the Netherlands; b Amsterdam Neuroscience, Compulsivity, Impulsivity & Attention program, Amsterdam, the Netherlands; c Erasmus University Medical Center, Rotterdam, the Netherlands; d The Generation R Study Group, Erasmus Medical Center, Rotterdam, the Netherlands; e Erasmus University Medical Center, Rotterdam, the Netherlands; f Harvard TH Chan School of Public Health, Boston, Massachusetts

**Keywords:** imaging, thalamus, cortical thickness, development, OCD

## Abstract

**Objective:**

Cross-sectional studies in children with obsessive-compulsive disorder (OCD) have found larger thalamic volume, which is not found at later ages. We previously found that 9- to 12-year-old children from the general population with clinical-level obsessive-compulsive symptoms (OCS) also have a larger thalamus. Thus, using a longitudinal design, we studied the relationship among thalamic volume, cortical maturation, and the course of OCS.

**Method:**

Children from the population-based Generation R Study underwent 1 or 2 (N = 2,552) magnetic resonance imaging (MRI) scans between the age of 9 and 16 years (baseline 9-12 years, follow-up 13-16 years). OCS were assessed with the Short Obsessive-Compulsive Disorder Screener (SOCS) questionnaire using both continuous and clinical cut-off measures to identify children with “probable OCD.” We applied linear regression models to investigate the cross-sectional relationship between brain morphology and OCS at age 13 to 16 years. Linear mixed-effect models were fitted to model the bidirectional longitudinal relationship between thalamus and OCS and the thalamus and cortical morphology.

**Results:**

Thalamic volume was not different between probable OCD cases and controls at age 13 to 16 years. Higher baseline thalamic volume predicted a relative persistence of OCS and a flatter slope of thinning in 12 cortical regions.

**Conclusion:**

Larger thalamic volume may be a subtle biomarker for persistent OCS symptoms. The persistence of OCS and cortical thickness in relation to earlier larger thalamic volume may reflect being at an earlier stage in neurodevelopment. Longitudinal designs with repeated multimodal brain imaging are warranted to improve our understanding of the neurodevelopmental processes underlying OCS and OCD.

**Study preregistration information:**

Relationship between obsessive-compulsive symptoms and brain morphology in school-aged children in the general population; https://osf.io/; y6vs2.

## Plain language summary

In this study utilizing Generation R data, 2,552 children from the general population underwent brain scans and assessment of obsessive-compulsive symptoms on 2 occasions between the ages of 10 and 16 years. The brain scans were used to measure the size and shape of various brain regions, including the thalamus and cortical morphology. Although there were no case-control differences, larger baseline thalamic volume predicted persistence of obsessive compulsive symptoms and a flatter slope of thinning of 12 cortical regions. These findings suggest that brain anatomy, and the thalamus in particular, may be a subtle biomarker for persistent symptoms that occur in obsessive-compulsive disorder.

Obsessive-compulsive disorder (OCD) affects approximately 1% to 2% of the population and often emerges during childhood.^1^^,^^2^ Whereas OCD is a clinical diagnosis, obsessive-compulsive symptoms (OCS) are common among children and constitute a part of typical development. It has been postulated that compulsive-like behaviors in typical development are a product of the simultaneous development of regions responsible for inhibitory control, combined with an increased need of reducing anxiety that results from growing demands on a child’s behavioral control in social contexts.^3^ These behaviors typically decrease during development, but persist in some children to an extent at which they develop clinical OCD.^4^ Albeit likely, it is not yet clear whether the OCS symptoms associated with typical development are on a continuum with clinical OCD. Neuroimaging provides a tool to study neurodevelopment and its relationship with the trajectory of OCS.

Morphological imaging studies have demonstrated that both pediatric OCD patients and 9- to 12-year-old children from the general population with clinical-level OCS have a larger thalamus compared to controls.^5^^,^^6^ In clinical samples these differences are age dependent, as larger thalamic volumes are not observed in adolescents and adult patients. In fact, adults with OCD have a smaller thalamus compared with controls.^7^ This suggests that increased thalamic volume may be an early marker of the disorder, most pronounced in unmedicated children with OCD, and disappears with continued development. Alternatively, this may reflect a difference in pathophysiology between very-early–onset cases and those with later onset, given that these results are based on cross-sectional studies and no longitudinal studies have been performed. Cortical abnormalities, including a thinner cortex in the parietal lobe, have also been shown in OCD patients compared with controls, which, in contrast to the thalamic findings, are more consistent across the lifespan.^8^ We did not replicate any of the cortical differences observed in clinical OCD in our analysis of 9- to 12-year-old children from the general population.^6^ However, children in the clinical studies were slightly older (mean age, 14 years) than children in our study (mean age 10 years). This failure to replicate may suggest that cortical abnormalities related to OCS appear later than thalamic abnormalities.

The exact mechanism behind the relationship between thalamic volume, cortical morphology, and OCD is unknown. Neural models have proposed that cortical abnormalities underlie impaired cognitive control of emotional responses, which in turn results in obsessions and compulsions characteristic of OCD.^9^ The thalamus and cortex have an intricate developmental relationship. Thalamocortical projections are formed during late embryonic development and are crucial for proper maturation of the cortical layer.^10^ Thalamic abnormalities have extensively been reported in schizophrenia, in which early thalamic dysfunction and subsequent improper prefrontal cortical maturation has been proposed as a mechanism for the development of its underlying cognitive deficits in attention, working memory, and cognitive flexibility, which overlap with OCD.^11^ The thalamocortical association and its role in psychopathology are further suggested by a recent genome-wide association study,^12^ which revealed a common genetic background between thalamic volume and numerous cortical areas that also overlapped with genetic loci for several psychiatric and neurological disorders. Genetic markers for OCD were not studied in that paper, but a different study found that genetic variants influencing thalamus volume were significantly associated with OCD risk,^13^ underlining a link between the thalamus, cortical development, and OCD.

Taken together, the literature underlines the intricate developmental relationship between the thalamus and cortex. Findings from clinical OCD and OCS within the general population suggest that differences in thalamic volume appear early but attenuate with age and may precede cortical alterations. We therefore hypothesized that thalamic volume during childhood (age 9-12 years) may predict change in cortical morphology between childhood (9-12 years) and adolescence (13-16 years).

A limitation of the current neuroimaging literature on OCD is that it consists of mostly cross-sectional studies, limiting the ability to determine the directionality of associations. Existing longitudinal studies linking brain morphology to OCD have primarily focused on morphological changes following treatment.^14^, ^15^, ^16^, ^17^, ^18^, ^19^, ^20^, ^21^ One study in particular found larger thalamic volume in pediatric OCD patients, with responders to paroxetine treatment showing a normalization of volume post-treatment.^14^ However, no population-based studies have investigated the longitudinal naturalistic relationship between brain morphology and OCS in children.

Within this context, we studied the relationship between thalamic volume, OCS, and cortical morphology in youth from the Generation R Study. First, we followed up on our previous study within this cohort in school-aged children (9-12 years),^6^ by conducting a similar cross-sectional analysis of subcortical and cortical morphology in relation to OCS in adolescents (13-16 years) of the Generation R Study. Next, we investigated the bidirectional longitudinal relationship between thalamic volume and OCS between 2 timepoints (9-12 years and 13-16 years). We also studied the relationship between baseline thalamic volume and cortical morphology over time to gain insight into the longitudinal association between the thalamus and cortex.

Given that clinical studies have repeatedly shown that increased thalamic volume is seen only in pediatric OCD patients and not in adolescents or adults, we hypothesized that the association between the thalamus and OCS would be less pronounced at age 13 to 16 years compared to our previous analysis at age 9 to 12 years. Furthermore, we expected that thalamic volume at baseline predicts symptoms at follow-up, and not vice versa. Finally, we hypothesized that thalamic volume at baseline predicts cortical alterations, specifically in regions that have previously been associated with OCD, and that these are related to the severity of OCS.

## Method

### Participants

Prior to conducting any analyses, we preregistered 2 analysis plans (https://osf.io/xmfpq and https://osf.io/azm5t), which we later combined into 1 study. This study was embedded within the Generation R Study, a prospective population-based cohort that tracks development from fetal life until early adulthood.^22^ Participants underwent a magnetic resonance imaging (MRI) scan at age 9 to 12 years (time 1, n = 3,992) and/or 13 to 16 years (time 2, n = 3,572).^23^ Participants were excluded in case of missing OCS data or unusable T_1_-weighted images due to poor registration, incidental findings, braces, or failed segmentation. The final sample size was dependent on the specific analysis performed (Figure S1 for flowchart). The study was approved by the Medical Ethical Committee of the institute and was conducted according to the Declaration of Helsinki.

### Short Obsessive-Compulsive Disorder Screener

OCS were assessed using the Short Obsessive-Compulsive Disorder Screener (SOCS).^24^ The SOCS is a 7-item measuring with a clinical cut-off score of 6 or greater and has a specificity of 0.84 and a sensitivity of 0.94 for detecting OCD in the general pediatric population.^24^ The group of children scoring above this clinical cut-off were named “probable OCD” in further analyses. We collected parent-rated (time 1 and 2) and self-report (time 2) scores. A weighted sumscore was calculated for those who had no more than 1 missing item on the SOCS. Children with more than 1 missing item were excluded from analyses. The self-report version was available only at time 2 and was limited to the first 5 items (Supplement 1, available online). Thus, for the self-report questionnaire, we used a weighted sumscore based on these 5 items only for the cross-sectional analyses.

### Imaging Acquisition and Processing

Structural MRI scans were collected using a 3T Discovery MR750w scanner (GE Healthcare, Waukesha, WI) using an 8-channel head coil. An inversion recovery fast spoiled gradient recalled sequence (IR-FSPGR) was used to obtain T_1_-weighted whole-brain images at 1-mm isotropic resolution. The sequence parameters were: GE option BRAVO, TR = 8.77 milliseconds, TE = 3.4 milliseconds, TI = 600 milliseconds, flip angle = 10°, matrix size = 220 × 220, field of view = 220 mm × 220 mm, number of slices = 230, ARC acceleration factor = 2. Automated preprocessing, subcortical segmentation, and cortical parcellation were performed using FreeSurfer version 6.0.1.^25^ To assess segmentation quality, each image was visually inspected for accuracy of the subcortical and cortical boundaries. We used the Desikan–Killiany atlas to extract thickness and surface area metrics from cortical regions-of-interest. We selected cortical regions-of-interest relevant to OCD by selecting 38 cortical regions (20 left, 18 right) that were significantly associated (before multiple comparisons correction) with OCD in a previous independent mega-analysis.^8^ Extracted regions included the following: mean left and right thalamus, caudal middle frontal (L + R), cuneus (L+R), fusiform (L + R), inferior parietal (L + R), inferior temporal (L), lateral occipital (L + R), lateral orbitofrontal (L + R), medial orbitofrontal (L + R), middle temporal (L + R), paracentral (L + R), parahippocampal (R), pars opercularis (L), pars triangularis (L), posterior cingulate (L + R), precuneus (L + R), rostral anterior cingulate (L), rostral middle frontal (L + R), superior frontal (R), superior parietal (L + R), superior temporal (L + R), supramarginal (L + R), and transverse temporal (L + R) (Table S1, available online). Total intracranial volume was estimated using the FreeSurfer-based Talairach transformation. Details on the FreeSurfer Quality assessments are outlined in Supplement 1, available online.

### Statistical Analyses

#### Cross-Sectional Analyses

All statistical analyses were performed in R, version 4.1.2.^26^ We first conducted a cross-sectional analysis at timepoint 2 (age 13-16) to compare with our previous findings conducted at timepoint 1 (age 9-12) and findings in adolescents from the ENIGMA-OCD consortium.^5^^,^^6^^,^^27^ In a post-hoc analysis, we investigated the group-by-age interaction of probable OCD children compared with symptom-free controls at both timepoints, to quantify the age-related differences between cases and controls (Supplement 1, available online).

Next, we compared thalamic volume, cortical thickness, and cortical surface area of probable OCD participants and matched symptom-free controls (Supplement 1, available online). We also performed a continuous analysis investigating the linear association between overall symptoms and brain outcomes. Thalamic volume was used as a single bilateral mean volume metric, whereas vertex-wise cortical thickness and surface area were used for the cortical analyses.

Because of the limited number of probable OCD cases at timepoint 2, we computed the Bayes Factor (BF_10_) to estimate the *a priori* likelihood of the null hypothesis (H0: no volume differences between probable OCD and controls) being true in case of negative finding, resulting in a number between 0 and 1 that is rated as follows: 1 (no evidence for H0), 0.33 to 1 (anecdotal evidence), 0.33 to 0.10 (moderate evidence), 0.10 to 0.03 (strong evidence), 0.03 to 0.01 (very strong evidence), and <0.01 (extreme evidence).^28^

### Longitudinal Analyses

We investigated the bidirectional longitudinal relationship between thalamic volume and OCS by applying linear mixed-effects models, modeling the within-subject change in OCS symptoms from baseline thalamic volume and vice versa (change in thalamic volume predicted by baseline symptoms). To investigate the longitudinal relationship between thalamic volume at baseline and change in cortical morphology, we applied a similar strategy. Linear mixed-effect models were run modeling the within-subject change in cortical thickness and cortical surface area on baseline thalamic volume. Models were adjusted for age, sex, ethnicity, maternal education level, maternal age at birth, and intracranial volume (Supplement 1, available online). In a sensitivity analysis, the thalamus–OCS association was additionally adjusted for general emotional and behavioral problems measured by the Child Behavior Checklist (CBCL),^29^ minus the CBCL Obsessive-Compulsive Scale (CBCL-OCS) items to avoid overcorrection.^30^ In the thalamo-cortical analyses, we additionally adjusted for total subcortical volume minus thalamic volume to adjust for global volume effects. To assess the specificity of the thalamo-cortical associations with respect to other subcortical structures, we conducted a post hoc comparison of baseline caudate and putamen volume predicting cortical morphology at follow-up.

We applied multiple imputation using chained equations (*mice*) to impute missing data of covariates.^31^ We applied a 2-tailed false discovery rate (Benjamini–Hochberg) correction to adjust for multiple comparisons. In the thalamus–OCS analysis, we corrected for the 2 directions that were tested. In the thalamo-cortical analyses, we corrected the significance threshold for multiple testing of 38 regions.

## Results

Sample characteristics are summarized in Table 1. The mean participant age was approximately 10 years during the first scan and 14 years during the second scan.

**Table 1:** Sample Characteristics

Characteristic	Time 1		Time 2	
	n	%	n	%
Total	3,170		2,068	
Male sex	1,575	50	946	46
Ethnicity				
Dutch	1,835	58	1,191	58
Non-Dutch	1,335	42	877	42
Maternal education level				
Low	218	7	162	8
Middle	1,303	41	874	42
High	1,649	52	1,032	50
	**Mean**	**SD**	**Mean**	**SD**
Age, y, at MRI scan	10.13	0.59	14.04	0.63
Maternal age, y, at birth	31.08	4.88	31.11	4.93
SOCS				
Parent-report	1.73	2.16	0.78	1.56
Probable OCD (%)^a^	6%		2%	
Self-report	—	—	3.28	2.78
CBCL^a^	15.68	13.92	17.21	15.71
Non-verbal IQ	102.70	14.91	102.47	14.59

Note: CBCL = Child Behavior Checklist; CBCL-OCD = Obsessive-Compulsive Subscale of the Child Behavior Checklist; MRI = magnetic resonance imaging; OCD = obsessive-compulsive disorder; SOCS = Short Obsessive-Compulsive Disorder Screener.

a Percentage of children with available MRI scan and SOCS data who scored above the clinical cutoff.

### Cross-Sectional Results

We found no cross-sectional association between thalamic volume and OCS at time 2 in either the case-control (Table S2, available online) or the analysis of continuous OCD traits (Table S3, available online). In post hoc analyses, we found no significant group-by-age interactions of probable OCD in relation to thalamic volume (Supplement 2, available online) across the age range from 9 to 16 years. For the case-control analyses, we computed that the Bayes factor was 0.196, to estimate the likelihood that there was indeed no difference in mean thalamic volume between cases and controls .

We also found no association between OCS, cortical thickness, and cortical surface area in the vertex-wise analyses at time 2.

### Longitudinal Relationship Between Thalamic Volume and OCS

**Figure 1:**
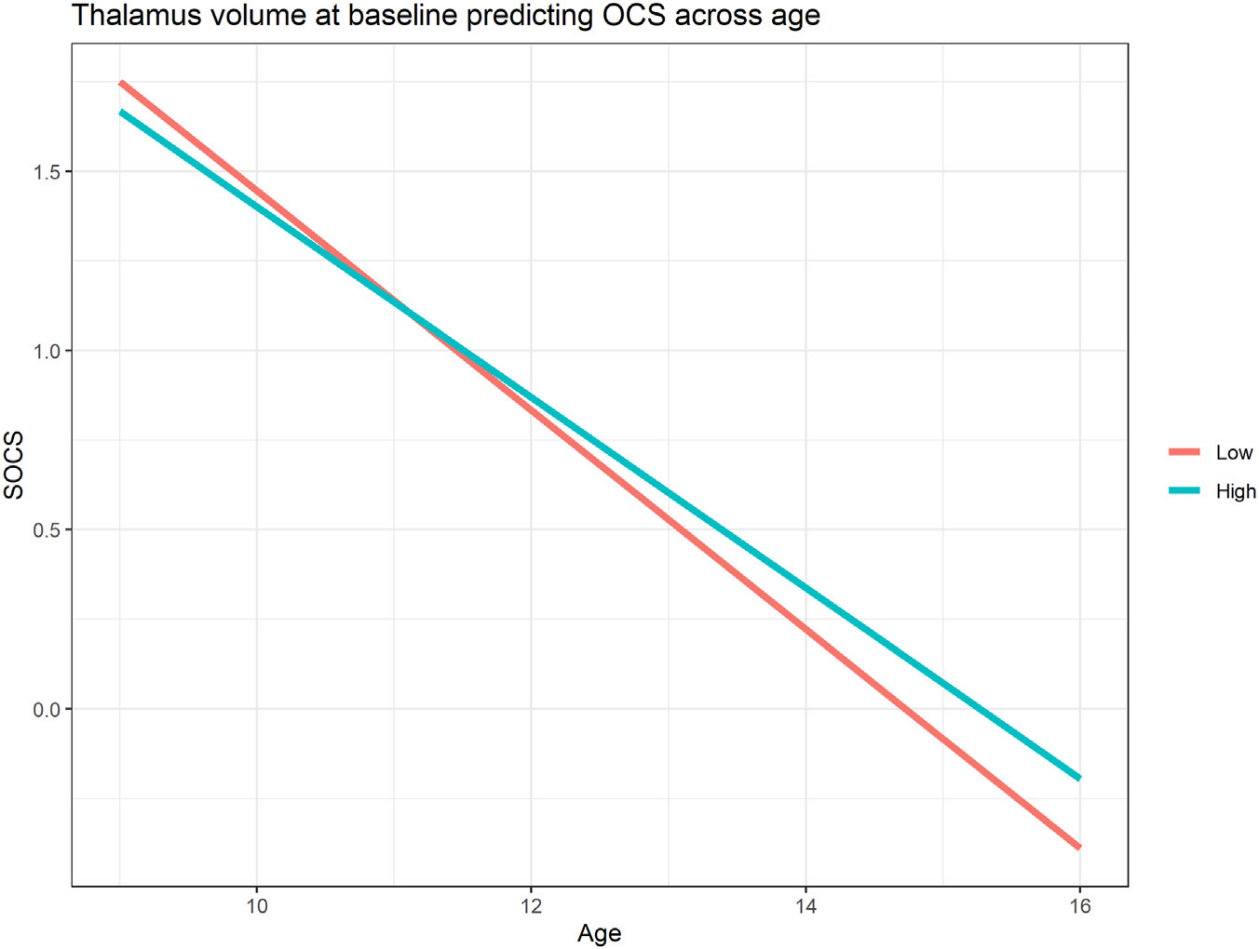
Visualization of the Predicted Model Estimates Derived From Linear Mixed-Effects Models for Obsessive-Compulsive Symptoms ***Note:****The y-axis represents the predicted Short Obsessive-Compulsive Disorder Screener (SOCS) scores based on model estimates, and the 2 lines (“low” and “high”) represent 1 SD below and above the mean thalamic volume.*

In linear mixed-effects models, higher thalamic volume at time 1 predicted a smaller decrease in OCS over time (β = 0.190, *p*_FDR_ = 0.028) after adjustment for covariates (Table 2, Figure 1). No significant associations were found between OCS at time 1 and change in thalamic volume over time (Table 3).

**Table 2:** Linear Mixed-Effects Models of Thalamic Volume at Baseline Predicting Change in Obsessive-Compulsive Symptoms

Term	Model	*β* (95% CI)	B	*t*	*p*	*p*_FDR_
Thal-by-age	M1	0.200 (0.028-0.372)	2.42E-05	2.275	.023	.028
	M2	0.190 (0.020-0.360)	2.31E-05	2.195	.028	.028

Note: Model 1 (M1) is adjusted for fixed effects, age at magnetic resonance imaging scan, sex, ethnicity, maternal education level, maternal age at birth, intracranial volume, and random effects of subject. Model 2 (M2) is additionally adjusted for emotional and behavioral problems. Model estimates represent the association between thalamic volume and change in parent-rated Short Obsessive-Compulsive Disorder Screener (SOCS) sumscores over time (thalamus-by-age interaction). *p*_FDR_ = False discovery rate–adjusted *p* value; thal-by-age = thalamus by age interaction.

**Table 3:** Linear Mixed-Effects Models of Obsessive-Compulsive Symptoms (OCS) at Baseline Predicting Change in Thalamic Volume

Term	Model	*β* (95% CI)	B	*t*	*p*	*p*_FDR_
OCS-by-age	M1	0.009 (–0.031 to 0.049)	0.253	0.433	.665	.665
	M2	0.009 (–0.031 to 0.049)	0.254	0.435	.663	.665

Note: Model 1 (M1) is adjusted for fixed effects, age at Short Obsessive-Compulsive Disorder Screener (SOCS) report, sex, ethnicity, maternal education level, maternal age at birth, intracranial volume, and random effects of subject. Model 2 (M2) is additionally adjusted for emotional and behavioral problems. Model estimates represent the association between parent-rated OCS and change in thalamic volume over time (OCS-by-age interaction). *p*_FDR_ = False discovery rate–adjusted *p* value.

### Longitudinal Relationship Between Thalamus and Cortical Morphology

Next, we investigated the longitudinal relationship between the thalamus and cortical maturation by testing whether thalamic volume at time 1 predicted the change in cortical morphology from time 1 to time 2. Using linear mixed-effects models, we found significant associations between baseline thalamic volume and change in cortical thickness over time in the bilateral fusiform, bilateral posterior cingulate, bilateral supramarginal, left paracentral, right middle temporal, right parahippocampal, right precuneus, right superior frontal, and right superior temporal cortices (significant results in Table 4; full results in Table S4, available online) after correction for multiple testing. The associations of the significant models all followed a similar pattern (Figure S2, available onine). Lower (compared with higher) thalamic volume at time 1 was associated with a steeper decrease in cortical thickness over time. Thalamic volume at time 1 did not predict change in cortical surface area in any of the cortical regions (Table S5, available online). Post hoc testing of the association between volumes of the putamen and caudate (at time 1) with changes in cortical thickness (time 1 to 2) revealed that the subcortical–cortical associations were not specific to the thalamus (Table S6, available online). We further explored the correlations between thalamus volume at time 1 and cortical thickness at both timepoints, respectively (Table S7, available online). To assess specificity, we also included correlations between global subcortical volume and cortical thickness. These correlations revealed a pattern similar to that of the linear mixed-model analysis, in which the correlations between thalamus and cortical thickness were different between timepoints, but these differences were not specific to thalamic volume.

**Table 4:** Linear Mixed-Effects Models of Thalamic Volume Predicting Cortical Thickness (Significant Results)

Cortical ROI	*β* (95% CI)	B	*t*	*P*	*p*_FDR_
Fusiform, cm^3^, (L)	0.375 (0.0727-0.677)	2.57E–06	2.43	.015	.048
Paracentral (L)	0.342 (0.0743-0.609)	2.80E–06	2.50	.012	.043
Posterior cingulate (L)	0.34 (0.104-0.577)	3.03E–06	2.82	.005	.021
Supramarginal (L)	0.313 (0.0566-0.569)	2.31E–06	2.39	.017	.049
Fusiform (R)	0.698 (0.403-0.992)	4.92E–06	4.64	3.78E–06	1.14E–04
Middle temporal (R)	0.814 (0.456-1.170)	7.19E–06	4.46	9.03E–06	1.14E–04
Parahippocampal (R)	0.332 (0.108-0.557)	4.16E–06	2.90	.004	.018
Posterior cingulate (R)	0.331 (0.0949-0.568)	2.79E–06	2.75	.006	.023
Precuneus (R)	0.401 (0.181-0.620)	2.95E–06	3.58	3.58E–04	.003
Superior frontal (R)	0.483 (0.168-0.798)	3.73E–06	3.00	.003	.015
Superior temporal (R)	0.528 (0.213-0.844)	4.52E–06	3.28	.001	.007
Supramarginal (R)	0.628 (0.357-0.898)	4.59E–06	4.54	6.11E–06	1.14E–04

Note: Models are adjusted for fixed effects, sex, ethnicity, maternal education level, maternal age at birth, intracranial volume, subcortical volume minus thalamic volume, and random effects of subject. Model estimates represent the association between thalamic volume and change in cortical thickness over time (thalamus-by-age interaction). L = left hemisphere; *p*_FDR_ = false discovery rate–adjusted *p* value; R = right hemisphere.

## Discussion

We investigated the cross-sectional and longitudinal relationship between thalamic volume and obsessive-compulsive symptoms, as well as between thalamic volume and cortical morphology and in youth from the population-based Generation R Study. In line with our hypotheses, we found that the previously reported larger thalamic volume in children with probable OCD at age 8 to 12 years is no longer present at age 13 to 16 years. Using vertex-wise analyses, we also found no relationship between cortical morphology and OCS at this age. The longitudinal analyses revealed that a larger thalamus at baseline was associated with a less steep decline in OCS symptoms over time. Furthermore, baseline thalamic volume predicted change in cortical thickness, but not cortical surface area, in 12 regions implicated in OCD, although this was not specific to the thalamus.

Whereas studies in children have found a larger thalamic volume in children with OCD, studies in adolescents and adults have not shown this finding.^5^^,^^7^ Thus, the relationship of larger thalamus and OCD should disappear over time, although the longitudinal nature of this trajectory has not been studied. Our negative cross-sectional findings are consistent with the hypothesis that larger thalamic volume in probable OCD (seen at 9-11 years of age) disappears by 13 to 15 years of age. Our post hoc calculation of a Bayes factor of 0.196 provides moderate evidence for no group differences in thalamic volumes in adolescents. In a post hoc analysis using the whole age range from 9 to 16 years modeled continuously, we did not find a probable-OCD by age interaction. This may suggest that the difference in the relation between OCD and thalamus between the 2 age groups may not be significant. Alternatively, the distribution of probable OCD across age may limit the power to detect significant interaction effects. Given the design of the study into 2 data collection waves, age follows a bimodal distribution around the 2 respective imaging waves with a relative sparsity of probable OCD cases at higher ages (Figure S3 for distribution plots).

We did find support for our hypothesis that baseline thalamic volume predicts OCS symptoms across time, and not vice versa. It is noteworthy to mention that thalamic volume at age 8 to 12 years was associated with a relative persistence of OCS across the whole sample, and not just in probable OCD. This implies that early thalamic volume has predictive capacity for youth with both clinical and subclinical symptoms in the general population. Thus, thalamic volume may serve as a potential marker for the development and course of OCD, although a follow-up with clinical diagnoses would be necessary to confirm this. A caveat to this is the modest strength of the association. However, Iglesias *et al.* have shown that the ability to predict clinical states improved after segmenting the thalamus into subregions.^32^ Furthermore, prediction could be improved by adding other established risk factors for OCD such as polygenic risk,^33^ perinatal complications,^34^ traumatic life events,^35^ or family history of OCD.^36^

The mechanism underlying the association between thalamic volume and OCS remains unknown. Given the intricate relationship between thalamic and cortical neurodevelopment,^10^ altered thalamic development may have an impact on development of the cortical layer. This is consistent with our finding that baseline thalamic volume predicted change in cortical thickness in 12 OCD-relevant regions. Thalamo-cortical projections start to emerge during late embryonic development and are crucial for the development of the cortical layer.^10^ The changes in quantity of these projections mirrors and preceded volumetric alterations in selected cortical regions.^37^ Developmental studies have shown that the thalamus expands in size during early childhood, followed by a steady decrease or stabilization throughout childhood and adolescence.^38^^,^^39^ Peak thalamic volume is reached later than in most cortical regions,^40^^,^^41^ which has been proposed to be driven by the significant fraction of total thalamic volume that contains myelinated fibers.^37^^,^^41^^,^^42^ White matter continues to be reshaped into adolescence and late adulthood, as myelination has been shown to not complete until the fourth decade of life.^43^ It was recently found that compulsivity was associated with reduced myelin-related growth in cortico-striatal regions in adolescents,^44^ suggesting that volumetric differences in the thalamus may be associated with altered development of white matter tracts connecting the thalamus to the cortex. Further evidence suggests that structural alterations underlying OCS may also translate to functional alterations involving the thalamus in both the general population^45^, ^46^, ^47^ and clinical samples.^48^ One clinical study found that decreased functional connectivity measured between the mediodorsal nucleus of the thalamus and dorsal anterior cingulate cortex was present only in pediatric OCD patients and not in adolescents and adults, suggesting that OCD-related functional alterations of the thalamus may be limited to pediatric patients.^48^ Using resting state functional MRI, we recently showed, in the sample used for the current analyses, that children endorsing at least 1 OCS, particularly hand washing, showed altered global network organization and altered network participation of thalamic nodes.^47^ A future step will be to investigate whether these network alterations also disappear at later ages.

We should note that the associations between baseline thalamic volume and cortical thickness were similarly observed between the caudate and putamen in the relation with thickness of a subset of cortical regions, suggesting more global than thalamus-specific associations. Furthermore, given that larger thalamic volume at baseline is associated with persistence of OCS, we had expected that larger thalamic volume would predict a steeper rather than flatter downward slope in cortical thickness. In our sample, thalamic size follows a linear downward trajectory with age. We speculate that a larger thalamus at baseline could reflect being at an earlier stage in neurodevelopment. Studies have reported nonlinear trajectories of cortical development.^49^ From that perspective, the differences in slope of the predicted linear trajectories of parahippocampal thickness may reflect different stages on the non-linear parahippocampal thickness trajectory. Intricately mapping neurodevelopment with multiple repeated measures is needed to test this hypothesis.

We did not find an association between thalamic volume and change in cortical surface area over time. In the ENIGMA-OCD analysis,^8^ changes in cortical surface area were found only in medicated children. This may suggest that surface area changes are the result of medication use. In the Generation R Study sample, most children are medication naive, and thus changes in surface area may appear only in medicated children.

Our study has several limitations. First, we investigated brain morphology and OCS at only 2 time points, between age 8 and 12 and between age 13 and 16 years. It has been well established that thalamic and cortical neurodevelopment follow a non-linear course.^38^^,^^39^^,^^49^^,^^50^ Investigating the neural correlates of OCS phenotypes during a period of complex neurodevelopment greatly benefits from a higher number of longitudinal measurements with short intervals. However, our study does provide important information showing the time period in which the thalamic differences disappeared during development. Second, the thalamus is a large structure; its volume provides information at the macroscale of the brain, but less about the cellular and subcellular processes that underly these volumetric changes. Future work can benefit from combining macroscale structural measures used here with other modalities such as fixel-based morphometry to gain more insight into white matter integrity of the connections between the thalamus and cortical areas. Multimodal studies also potentially improve predictive power for OCS by extracting more relevant information from the brain. These approaches should be supported by ex vivo or animal studies to investigate morphological changes at the histological scale. There were also some limitations in the SOCS data. The parent-rated scale was available at both timepoints, whereas a reduced 5-item self-report was available only at the second timepoint. The number of probable OCD children was lower at the second timepoint (2%) than at the first timepoint (6%). This reduction may suggest that parents are less able to assess OCS symptoms during adolescence, a period of time when youth often spend less time with family and more time with peers. The SOCS items assess mostly observable behaviors associated with OCS (eg, hand washing and checking behaviors), which the parents may be less likely to observe in teens due to less time spent with family or, alternatively, greater cognitive development may help mask the symptoms. Alternatively, the higher percentage of probable OCD at younger ages may be partly explained by younger children still displaying OCS as a result of typical development. Because OCS are not typically seen during typical development in early adolescence, the percentage of probable OCD may be closer to the prevalence of clinical OCD at that age. We considered supplementing our results using the Obsessive-Compulsive Subscale of the Child Behavior Checklist (CBCL-OCS). However, the correlation between CBCL-OCS and SOCS was not strong (0.37). Even though the CBCL-OCS has been widely used in studies of OCS, several items on the scale do not specifically address symptoms of OCD, such as “feels too guilty,” “worries,” “strange behavior,” “strange ideas.” We therefore focused on the SOCS over the CBCL-OCS. Finally, although our study has the benefit of studying OCS at the population-level, the Generation R Study does not provide information on clinical diagnoses. Therefore, despite using an instrument with excellent test characteristics,^24^ we are not able to confirm that the children identified as having “probable OCD” have clinical OCD. In addition, although we know that medication use in the Generation R sample is low, our records of exposure to medications is limited. Future waves of the Generation R Study will include a diagnostic interview, enabling the combination of more repeated imaging measurements with information on clinical diagnoses, thereby addressing some of the previous limitations.

In conclusion, the current study confirms our hypothesis that thalamic enlargement in relation to probable OCD in the general population differs in adolescence, and that thalamic volume predicts change in OCS, rather than OCS symptoms predicting thalamic volume. Furthermore, we showed that thalamic volume and probable OCD at age 8 to 12 years is associated with thinning of OCD-relevant cortical regions. The mechanisms underlying the observed associations between the thalamus, cortex, and OCS may include altered myelination of thalamocortical tracts or an overall altered pace of neurodevelopment.
