# Supplementary material for: The Longitudinal Relationship Between Brain Morphology and Obsessive-Compulsive Symptoms in Children From the General Population
Source: JAACAP Open. 2023 Dec 25;2(2):126–34. doi: 10.1016/j.jaacop.2023.11.003 (PMC11562553; doi:10.1016/j.jaacop.2023.11.003)
Supplement: Supplemental Data [file mmc1.docx]

**SUPPLEMENTAL INFORMATION**

**Supplement 1**

S1.1 Items of Short Obsessive-Compulsive Disorder Screener (SOCS)

| **N** | **Item** | **Self**  **report** | **Parent**  **rated** |
| --- | --- | --- | --- |
| 1 | Does your child often check or count things or touch them – even though he/she knows that this is not really necessary? | T2 | T1, T2 |
| 2 | Is your child very concerned about keeping his/her hands clean? | T2 | T1, T2 |
| 3 | Does your child have to do things a certain number of times before he/she feels that it is right? | T2 | T1, T2 |
| 4 | Does your child sometimes have difficulty finishing homework or  chores because he/she has to do things again and again? | T2 | T1, T2 |
| 5 | Is your child very concerned if he/she did not do something  exactly the way he/she wanted to? | T2 | T1, T2 |
| 6 | Does this cause any problems for him/her? | - | T1, T2 |
| 7 | Does he/she attempt to stop this? | - | T1, T2 |

Note: Columns ‘self report’ and ‘parent rated’ represent at which timepoint (T1 = time 1; T2 = time 2) each item is present.

S1.2 Freesurfer Quality assessments

The cortical parcellations were evaluated by how well the boundaries between pial and white matter surfaces were delineated by FreeSurfer. The temporal and frontal lobe quality was assessed separately since these structures are captured less accurately more often. Scans were rated as either ‘usable’ or ‘ unusable’ by a team of six different raters that underwent a protocolized training. Scans were divided across raters, ensuring that each scan was rated by two raters independently. Only scans rated as usable by both raters were included.

For quality checking of the thalamus segmentations, we first determined statistical outliers by calculating the interquartile range for each of the volumes. Biologically implausible extreme values would be excluded, but this was not the case in any segmentations. Thalamus segmentations were visually inspected by two trained raters for each scan. Segmentations were rated poor, questionable or good. If rated good, the structure was included. If rated poor, the structure was excluded. Any segmentations rated questionable were evaluated again by both raters and made a final decision together.

S1.3 Statistical analyses

*Cross-sectional analyses*

To model the cross-sectional relationship between thalamus volume and OCS at time 2 we used the parent-reported and self-reported SOCS score to compare thalamus volume between children with probable OCD (SOCS ≥ 6) and symptom-free controls matched on age, sex and ethnicity and maternal education level. Thalamus volumes were residualized for intracranial volume (ICV) to adjust for the effects of head size. We also conducted continuous analyses by running multiple linear regression models with SOCS sumscores as independent variable and thalamus volume as dependent variable, adjusted for age, sex, ethnicity, maternal education level and ICV. We studied the cross-sectional relationship between cortical morphology and OCS at time 2 by running vertex-wise analyses using the QDECR package (<https://www.qdecr.com/>).

As a posthoc analysis to the probable OCD case-control analysis, we conducted a probable-OCD-by-age interaction analysis using data from two timepoints. We leveraged the measurements at both timepoints to form a larger cross-sectional dataset of unique participants with a broader age range that covers both timepoints. We selected the participants with one measurement (time 1 or time 2) and one random observation of the participaints with two measurement (time 1 and time 2). This resulted in a combined dataset of 1961 participants, of which 177 were in the ‘probable OCD’ group. We applied linear regression modelling the binary ‘probable OCD’ and age interaction, adjusting for sex, ethnicity, maternal education level and intracranial volume. Results are presented below in S3.

*Linear mixed model analyses modelling the relationship between thalamus and OCS*

In longitudinal analyses we modelled the bidirectional longitudinal relationship between thalamus volume and OCS. More specifically, we applied a linear mixed-effects model to assess whether baseline thalamus volume predicts change in OCS and whether baseline OCS predicts change in thalamus volume. The regression equation is depicted below.

Baseline thalamus volume predicting change in OCS

SOCS*_ij_* ~ age-SOCS*_ij_* + age-MRI*_i_* + MRI*_i_* + **MRI*_i_* *x* age-SOCS*_ij_*** + ICV*_i_* + covariates + (1 | participant).

In this equation, *i* represent participant and *j* time point. Models were adjusted for age, sex, ethnicity, maternal education level, maternal age at birth and ICV. The ‘(1 | participant)’ term is the random intercept for participant, which is used to adjust for the baseline value. The coefficient of interested is the **bold** interaction term ‘ **MRI*_i_* *x* age-SOCS*_ij_’****,* which captures the effect of time the association between baseline thalamus and OCS (i.e. baseline thalamus predicting change in OCS score).

**Supplement 2**

*S2.1 Posthoc cross-sectional analyses*

The expanded group with unique participants spanning both timepoints included 1961 participants, of which 177 were within the probable OCD group (142 timepoint 1; 35 timepoint 2), see Figure S1.

In the models investigating the interaction between probable OCD in age on thalamus volume, we found no significant probable-OCD-by-age interaction (B_OCD-by-age_ = -4.90, t = -0.209, *p* = 0.834) after adjustment for age, sex, maternal ethnicity, maternal education level and intracranial volume. We additionally did not find a significant interaction with probable OCD and Age-squared (B_OCD-by-agesqrd_ = -0.188, t = -0.187, *p* = 0.851).

**SUPPLEMENTAL TABLES & FIGURES**

**Table S1: List of cortical regions of interest used in analyses**

| Caudal middle frontal (L) |
| --- |
| Cuneus (L) |
| Fusiform (L) |
| Inferior parietal (L) |
| Inferior temporal (L) |
| Lateral occipital (L) |
| Lateral orbitofrontal (L) |
| Medial orbitofrontal (L) |
| Middle temporal (L) |
| Paracentral (L) |
| Pars opercularis (L) |
| Parts triangularis (L) |
| Posterior cingulate (L) |
| Precuneus (L) |
| Rostral anterior cingulate (L) |
| Rostral middle frontal (L) |
| Superior parietal (L) |
| Superior temporal (L) |
| Supramarginal (L) |
| Transverse temporal (L) |
| Caudal middle frontal (R) |
| Cuneus (R) |
| Fusiform (R) |
| Inferior parietal (R) |
| Lateral occipital (R) |
| Lateral orbitofrontal (R) |
| Medial orbitofrontal (R) |
| Middle temporal (R) |
| Paracentral (R) |
| Parahippocampal (R) |
| Posterior cingulate (R) |
| Precuneus (R) |
| Rostral middle frontal (R) |
| Superior frontal (R) |
| Superior parietal (R) |
| Superior temporal (R) |
| Supramarginal (R) |
| Transverse temporal (R) |

**Table S2: Cross-sectional difference in thalamus volume between probable OCD and controls**

| Reporter | Model | *d* [95% CI] | t | *p* | *p*_FDR_ |
| --- | --- | --- | --- | --- | --- |
| Parent-report | Model 1 | -0.023 [-0.474, 0.428] | -0.101 | 0.920 | 0.920 |
|  | Model 2 | 0.014 [-0.437, 0.465] | 0.061 | 0.952 | 0.952 |
| Self-report | Model 1 | 0.044 [-0.149, 0.238] | 0.453 | 0.651 | 0.920 |
|  | Model 2 | 0.058 [-0.136, 0.251] | 0.442 | 0.659 | 0.952 |

Table S2: groups were matched on age, sex, ethnicity, maternal education level. Model 1 is adjusted for intracranial volume. Model 2 is additionally adjusted for emotional and behavioural problems. *d =* Cohen’s d; 95% CI = 95% confidence interval; *p*_FDR_ = False Discovery Rate-adjusted p value.

**Table S3: Continuous association between thalamus volume and obsessive-compulsive symptoms**

| Reporter | Model | B [95% CI] | t | *p* | *p*_FDR_ |
| --- | --- | --- | --- | --- | --- |
| Parent-report | Model 1 | -6.62 [-21.66, 8.43] | -0.862 | 0.389 | 0.672 |
|  | Model 2 | -3.29 [-19.19, 12.61] | -0.406 | 0.685 | 0.689 |
| Self-report | Model 1 | 0.95 [-7.65, 9.55] | 0.216 | 0.829 | 0.799 |
|  | Model 2 | 1.83 [-6.93, 10.59] | 0.410 | 0.682 | 0.689 |

Table S3: Model 1 is adjusted age, sex, ethnicity, maternal education level and intracranial volume. Model 2 is additionally adjusted for emotional and behavioural problems. *d =* Cohen’s d; 95% CI = 95% confidence interval; *p*_FDR_ = False Discovery Rate-adjusted p value.

**Table S4: Linear mixed-effects models of thalamus volume predicting cortical thickness**

| **Cortical ROI** | **β [95% CI]** | **B** | ***t*** | ***p*** | ***p_FDR_*** |
| --- | --- | --- | --- | --- | --- |
| Caudal middle frontal (L) | 0.335 [0.0108, 0.659] | 2.64E-06 | 2.03 | 0.043 | 0.092 |
| Cuneus (L) | -0.024 [-0.263, 0.215] | -2.20E-07 | -0.197 | 0.844 | 0.921 |
| Fusiform (L) | 0.375 [0.0727, 0.677] | 2.57E-06 | 2.43 | 0.0152 | 0.048 |
| Inferior parietal (L) | 0.304 [-0.0408, 0.649] | 2.70E-06 | 1.73 | 0.0842 | 0.16 |
| Inferior temporal (L) | 0.356 [0.0106, 0.702] | 2.96E-06 | 2.02 | 0.0436 | 0.092 |
| Lateral occipital (L) | -0.0153 [-0.344, 0.313] | -1.04E-07 | -0.0912 | 0.927 | 0.952 |
| Lateral orbitofrontal (L) | 0.284 [-0.0147, 0.583] | 2.68E-06 | 1.86 | 0.0626 | 0.125 |
| Medial orbitofrontal (L) | 0.157 [-0.154, 0.468] | 1.57E-06 | 0.989 | 0.323 | 0.423 |
| Middle temporal (L) | 0.22 [-0.0835, 0.523] | 2.00E-06 | 1.42 | 0.156 | 0.237 |
| Paracentral (L) | 0.342 [0.0743, 0.609] | 2.80E-06 | 2.5 | 0.0124 | 0.0428 |
| Pars opercularis (L) | 0.301 [0.0374, 0.564] | 2.31E-06 | 2.24 | 0.0253 | 0.0602 |
| Parts triangularis (L) | 0.2 [-0.0653, 0.464] | 1.68E-06 | 1.48 | 0.14 | 0.222 |
| Posterior cingulate (L) | 0.34 [0.104, 0.577] | 3.03E-06 | 2.82 | 4.94E-03 | 0.0208 |
| Precuneus (L) | 0.258 [0.0395, 0.476] | 1.85E-06 | 2.32 | 0.0208 | 0.0526 |
| Rostral anterior cingulate (L) | 0.214 [-0.0654, 0.494] | 2.68E-06 | 1.5 | 0.133 | 0.22 |
| Rostral middle frontal (L) | 0.0803 [-0.217, 0.377] | 6.28E-07 | 0.53 | 0.596 | 0.731 |
| Superior parietal (L) | 0.252 [-0.0557, 0.559] | 1.73E-06 | 1.61 | 0.109 | 0.188 |
| Superior temporal (L) | 0.186 [-0.0875, 0.459] | 1.48E-06 | 1.33 | 0.183 | 0.258 |
| Supramarginal (L) | 0.313 [0.0566, 0.569] | 2.31E-06 | 2.39 | 0.0169 | 0.0493 |
| Transverse temporal (L) | 0.0334 [-0.193, 0.259] | 4.03E-07 | 0.289 | 0.772 | 0.89 |
| Caudal middle frontal (R) | 0.207 [-0.103, 0.516] | 1.60E-06 | 1.31 | 0.191 | 0.26 |
| Cuneus (R) | -0.0255 [-0.287, 0.236] | -2.21E-07 | -0.191 | 0.849 | 0.921 |
| Fusiform (R) | 0.698 [0.403, 0.992] | 4.92E-06 | 4.64 | 3.78E-06 | 1.14E-04 |
| Inferior parietal (R) | 0.195 [-0.0883, 0.479] | 1.51E-06 | 1.35 | 0.177 | 0.258 |
| Lateral occipital (R) | 0.0193 [-0.29, 0.328] | 1.36E-07 | 0.122 | 0.903 | 0.952 |
| Lateral orbitofrontal (R) | 0.631 [0.264, 0.998] | 5.39E-06 | 3.37 | 7.75E-04 | 5.89E-03 |
| Medial orbitofrontal (R) | 0.0805 [-0.255, 0.416] | 8.31E-07 | 0.471 | 0.638 | 0.758 |
| Middle temporal (R) | 0.814 [0.456, 1.17] | 7.19E-06 | 4.46 | 9.03E-06 | 1.14E-04 |
| Paracentral (R) | 0.229 [-0.0394, 0.498] | 1.86E-06 | 1.67 | 0.0946 | 0.171 |
| Parahippocampal (R) | 0.332 [0.108, 0.557] | 4.16E-06 | 2.9 | 3.76E-03 | 0.0179 |
| Posterior cingulate (R) | 0.331 [0.0949, 0.568] | 2.79E-06 | 2.75 | 6.13E-03 | 0.0233 |
| Precuneus (R) | 0.401 [0.181, 0.62] | 2.95E-06 | 3.58 | 3.58E-04 | 0.0034 |
| Rostral middle frontal (R) | 0.144 [-0.195, 0.484] | 1.07E-06 | 0.832 | 0.406 | 0.514 |
| Superior frontal (R) | 0.483 [0.168, 0.798] | 3.73E-06 | 3 | 2.72E-03 | 0.0148 |
| Superior parietal (R) | 0.000125 [-0.316, 0.316] | 8.54E-10 | 0.000774 | 0.999 | 0.999 |
| Superior temporal (R) | 0.528 [0.213, 0.844] | 4.52E-06 | 3.28 | 1.06E-03 | 6.73E-03 |
| Supramarginal (R) | 0.628 [0.357, 0.898] | 4.59E-06 | 4.54 | 6.11E-06 | 1.14E-04 |
| Transverse temporal (R) | 0.298 [0.0462, 0.549] | 3.50E-06 | 2.32 | 0.0205 | 0.0526 |

Table S4: Models are adjusted for fixed effects, sex, ethnicity, maternal education level, intracranial volume, subcortical volume minus thalamus volume and random effects of subject. Model estimates represent the association between thalamus volume and change in cortical thickness over time (thalamus-by-age interaction). 95% CI = 95% confidence interval; L = left hemisphere; pFDR = False-Discovery-Rate-adjusted p-value; R = right hemisphere.

**Table S5: Linear mixed-effects models of thalamus volume predicting cortical surface area**

| **Cortical ROI** | **β [95% CI]** | **B** | ***t*** | ***p*** | ***p_FDR_*** |
| --- | --- | --- | --- | --- | --- |
| Caudal middle frontal (L) | -0.00568 [-0.15, 0.139] | -1.39E-04 | -0.0771 | 0.939 | 0.964 |
| Cuneus (L) | 0.00746 [-0.139, 0.153] | 1.03E-04 | 0.1 | 0.92 | 0.964 |
| Fusiform (L) | -0.0518 [-0.188, 0.0842] | -1.27E-03 | -0.747 | 0.455 | 0.641 |
| Inferior parietal (L) | -0.155 [-0.311, 0.0013] | -6.88E-03 | -1.94 | 0.0522 | 0.22 |
| Inferior temporal (L) | 0.131 [-0.0434, 0.306] | 4.20E-03 | 1.47 | 0.141 | 0.383 |
| Lateral occipital (L) | 0.00639 [-0.126, 0.139] | 3.07E-04 | 0.0945 | 0.925 | 0.964 |
| Lateral orbitofrontal (L) | -0.0995 [-0.351, 0.152] | -2.03E-03 | -0.776 | 0.438 | 0.64 |
| Medial orbitofrontal (L) | 0.155 [-0.173, 0.483] | 2.42E-03 | 0.927 | 0.354 | 0.608 |
| Middle temporal (L) | 0.0615 [-0.0909, 0.214] | 1.67E-03 | 0.791 | 0.429 | 0.64 |
| Paracentral (L) | -0.112 [-0.254, 0.0296] | -1.16E-03 | -1.55 | 0.121 | 0.354 |
| Pars opercularis (L) | -0.0266 [-0.158, 0.105] | -4.35E-04 | -0.396 | 0.692 | 0.792 |
| Parts triangularis (L) | -0.0766 [-0.222, 0.0689] | -9.94E-04 | -1.03 | 0.302 | 0.574 |
| Posterior cingulate (L) | -0.135 [-0.27, 0.000235] | -1.47E-03 | -1.96 | 5.06E-02 | 0.22 |
| Precuneus (L) | -0.111 [-0.242, 0.0201] | -3.49E-03 | -1.66 | 0.0974 | 0.336 |
| Rostral anterior cingulate (L) | -0.106 [-0.295, 0.0825] | -1.11E-03 | -1.1 | 0.27 | 0.571 |
| Rostral middle frontal (L) | -0.0995 [-0.288, 0.0889] | -5.15E-03 | -1.03 | 0.301 | 0.574 |
| Superior parietal (L) | -0.162 [-0.332, 0.00786] | -7.78E-03 | -1.87 | 0.0618 | 0.235 |
| Superior temporal (L) | -0.0498 [-0.162, 0.0628] | -1.46E-03 | -0.867 | 0.386 | 0.611 |
| Supramarginal (L) | -0.0779 [-0.193, 0.0372] | -3.48E-03 | -1.33 | 0.185 | 0.439 |
| Transverse temporal (L) | -0.0369 [-0.168, 0.0944] | -1.56E-04 | -0.551 | 0.582 | 0.727 |
| Caudal middle frontal (R) | -0.0634 [-0.2, 0.0732] | -1.52E-03 | -0.909 | 0.363 | 0.608 |
| Cuneus (R) | -0.122 [-0.27, 0.0259] | -1.82E-03 | -1.62 | 0.106 | 0.336 |
| Fusiform (R) | 0.0839 [-0.0559, 0.224] | 2.04E-03 | 1.18 | 2.40E-01 | 5.35E-01 |
| Inferior parietal (R) | -0.0241 [-0.151, 0.102] | -1.19E-03 | -0.374 | 0.709 | 0.792 |
| Lateral occipital (R) | 0.0275 [-0.0935, 0.148] | 1.32E-03 | 0.445 | 0.656 | 0.779 |
| Lateral orbitofrontal (R) | -0.324 [-0.622, -0.026] | -7.44E-03 | -2.13 | 3.33E-02 | 2.20E-01 |
| Medial orbitofrontal (R) | -0.322 [-0.583, -0.0613] | -4.99E-03 | -2.42 | 0.0156 | 0.198 |
| Middle temporal (R) | 0.0416 [-0.111, 0.194] | 1.18E-03 | 0.535 | 5.93E-01 | 7.27E-01 |
| Paracentral (R) | -0.166 [-0.299, -0.0339] | -2.03E-03 | -2.46 | 0.014 | 0.198 |
| Parahippocampal (R) | 0.22 [0.0155, 0.424] | 1.07E-03 | 2.11 | 3.52E-02 | 0.22 |
| Posterior cingulate (R) | -0.128 [-0.25, -0.00559] | -1.44E-03 | -2.05 | 4.06E-02 | 0.22 |
| Precuneus (R) | -0.124 [-0.233, -0.0158] | -4.16E-03 | -2.25 | 2.49E-02 | 0.22 |
| Rostral middle frontal (R) | -0.0592 [-0.226, 0.108] | -3.42E-03 | -0.694 | 0.488 | 0.662 |
| Superior frontal (R) | -0.206 [-0.361, -0.05] | -1.21E-02 | -2.59 | 9.73E-03 | 0.198 |
| Superior parietal (R) | -0.0498 [-0.213, 0.114] | -2.20E-03 | -0.597 | 0.551 | 0.722 |
| Superior temporal (R) | 0.00271 [-0.129, 0.135] | 6.93E-05 | 0.0403 | 9.68E-01 | 9.68E-01 |
| Supramarginal (R) | -0.0897 [-0.217, 0.038] | -3.17E-03 | -1.38 | 1.69E-01 | 4.28E-01 |
| Transverse temporal (R) | -0.0745 [-0.237, 0.0876] | -2.20E-04 | -0.901 | 0.368 | 0.608 |

Table S5: Models are adjusted for fixed effects, sex, ethnicity, maternal education level, intracranial volume, subcortical volume minus thalamus volume and random effects of subject. Model estimates represent the association between thalamus volume and change in cortical surface area over time (thalamus-by-age interaction). 95% CI = 95% confidence interval; L = left hemisphere; pFDR = False-Discovery-Rate-adjusted p-value; R = right hemisphere.

**Table S6: Linear mixed-effects models of subcortical volumes predicting cortical thickness**

| **Cortical ROI** | **Volume** | **β [95% CI]** | **B** | ***t*** | ***p*** | ***p_FDR_*** |
| --- | --- | --- | --- | --- | --- | --- |
| Fusiform (L) | Caudate | 0.111 [-0.136, 0.358] | 1.30E-06 | 0.88 | 0.377 | 0.404 |
|  | Putamen | 0.508 [0.239, 0.777] | 4.71E-06 | 3.70 | 0.000 | **0.001** |
|  | Thalamus | 0.375 [0.073, 0.677] | 2.57E-06 | 2.43 | 0.015 | **0.048** |
| Paracentral (L) | Caudate | 0.138 [-0.080, 0.355] | 1.92E-06 | 1.24 | 0.215 | 0.248 |
|  | Putamen | 0.372 [0.134, 0.610] | 4.13E-06 | 3.07 | 0.002 | **0.004** |
|  | Thalamus | 0.342 [0.074, 0.609] | 2.80E-06 | 2.50 | 0.012 | **0.043** |
| Posterior Cingulate (L) | Caudate | 0.278 [0.084, 0.471] | 4.20E-06 | 2.81 | 0.005 | **0.020** |
|  | Putamen | 0.504 [0.293, 0.714] | 6.06E-06 | 4.68 | 3.19E-06 | **1.19E-05** |
|  | Thalamus | 0.340 [0.104, 0.577] | 3.03E-06 | 2.82 | 0.005 | **0.021** |
| Supramarginal (L) | Caudate | 0.146 [-0.062, 0.355] | 1.84E-06 | 1.37 | 0.170 | 0.231 |
|  | Putamen | 0.319 [0.091, 0.547] | 3.19E-06 | 2.74 | 0.006 | **0.009** |
|  | Thalamus | 0.313 [0.057, 0.569] | 2.31E-06 | 2.39 | 0.017 | **0.049** |
| Fusiform (R) | Caudate | 0.341 [0.099, 0.582] | 4.09E-06 | 2.76 | 0.006 | **0.020** |
|  | Putamen | 0.660 [0.396, 0.923] | 6.30E-06 | 4.91 | 1.04E-06 | **5.20E-06** |
|  | Thalamus | 0.698 [0.403, 0.992] | 4.92E-06 | 4.64 | 3.78E-06 | **1.14E-04** |
| Middle Temporal (R) | Caudate | 0.433 [0.140, 0.727] | 6.52E-06 | 2.89 | 0.004 | **0.020** |
|  | Putamen | 0.568 [0.246, 0.890] | 6.79E-06 | 3.46 | 0.001 | **0.001** |
|  | Thalamus | 0.814 [0.456, 1.170] | 7.19E-06 | 4.46 | 9.03E-06 | **1.14E-04** |
| Parahippocampal (R) | Caudate | 0.237 [0.054, 0.420] | 5.05E-06 | 2.53 | 0.011 | **0.024** |
|  | Putamen | 0.219 [0.018, 0.420] | 3.71E-06 | 2.14 | 0.033 | **0.033** |
|  | Thalamus | 0.332 [0.108, 0.557] | 4.16E-06 | 2.90 | 0.004 | **0.018** |
| Posterior Cingulate (R) | Caudate | 0.290 [0.097, 0.483] | 4.15E-06 | 2.94 | 0.003 | **0.020** |
|  | Putamen | 0.287 [0.076, 0.499] | 3.27E-06 | 2.66 | 0.008 | **0.010** |
|  | Thalamus | 0.331 [0.095, 0.568] | 2.79E-06 | 2.75 | 0.006 | **0.023** |
| Precuneus (R) | Caudate | 0.242 [0.063, 0.421] | 3.04E-06 | 2.65 | 0.008 | **0.020** |
|  | Putamen | 0.640 [0.448, 0.833] | 6.39E-06 | 6.51 | 1.12E-10 | **1.65E-09** |
|  | Thalamus | 0.401 [0.181, 0.620] | 2.95E-06 | 3.58 | 3.58E-04 | **0.003** |
| Superior frontal (R) | Caudate | 0.171 [-0.087, 0.430] | 2.26E-06 | 1.30 | 0.194 | 0.242 |
|  | Putamen | 0.328 [0.046, 0.611] | 3.44E-06 | 2.28 | 0.023 | **0.024** |
|  | Thalamus | 0.483 [0.168, 0.798] | 3.73E-06 | 3.00 | 0.003 | **0.015** |
| Superior temporal (R) | Caudate | 0.202 [-0.056, 0.461] | 2.95E-06 | 1.53 | 0.126 | 0.189 |
|  | Putamen | 0.398 [0.115, 0.681] | 4.61E-06 | 2.76 | 0.006 | **0.009** |
|  | Thalamus | 0.528 [0.213, 0.844] | 4.52E-06 | 3.28 | 0.001 | **0.007** |
| Supramarginal (R) | Caudate | 0.301 [0.079, 0.522] | 3.75E-06 | 2.66 | 0.008 | **0.020** |
|  | Putamen | 0.613 [0.372, 0.854] | 6.07E-06 | 4.99 | 6.97E-07 | **5.20E-06** |
|  | Thalamus | 0.628 [0.357, 0.898] | 4.59E-06 | 4.54 | 6.11E-06 | **1.14E-04** |

Table S6: Models are adjusted for fixed effects, sex, ethnicity, maternal education level, intracranial volume, total subcortical volume minus the volume of the predictor, and random effects of subject. Model estimates represent the association between volume of the subcortical region and change in cortical thickness over time (volume-by-age interaction). 95% CI = 95% confidence interval; L = left hemisphere; pFDR = False-Discovery-Rate-adjusted p-value; R = right hemisphere.

**Table S7: Pearson correlations between thalamus volume, subcortical volume and cortical thickness**

|  | Thal T1 –  Cortical T1 | Thal T1 – Cortical T2 | Subcortical T1 – Cortical T1 | Subcortical T1 – Cortical T2 |
| --- | --- | --- | --- | --- |
| Caudal middle frontal (L) | -0.02 | 0.05 | 0 | 0.06 |
| Cuneus (L) | 0.11 | 0.09 | 0.07 | 0.13 |
| Fusiform (L) | 0.01 | 0.06 | 0.02 | 0.09 |
| Inferior parietal (L) | 0.02 | 0.08 | 0.03 | 0.14 |
| Inferior temporal (L) | -0.01 | 0.05 | -0.03 | 0.04 |
| Lateral occipital (L) | 0.15 | 0.18 | 0.12 | 0.19 |
| Lateral orbitofrontal (L) | -0.1 | -0.04 | -0.01 | 0.03 |
| Medial orbitofrontal (L) | -0.06 | -0.05 | -0.03 | 0.02 |
| Middle temporal (L) | -0.06 | -0.02 | -0.04 | 0.02 |
| Paracentral (L) | 0 | 0.09 | -0.01 | 0.1 |
| Pars opercularis (L) | 0.01 | 0.07 | 0 | 0.05 |
| Parts triangularis (L) | -0.03 | 0.01 | -0.01 | -0.01 |
| Posterior cingulate (L) | -0.08 | -0.04 | -0.07 | 0.01 |
| Precuneus (L) | 0.07 | 0.16 | 0.07 | 0.17 |
| Rostral anterior cingulate (L) | -0.16 | -0.15 | -0.08 | 0.01 |
| Rostral middle frontal (L) | -0.02 | 0.01 | 0 | 0.03 |
| Superior parietal (L) | 0.03 | 0.1 | 0.06 | 0.16 |
| Superior temporal (L) | 0 | 0.04 | 0.06 | 0.09 |
| Supramarginal (L) | -0.02 | 0.07 | -0.01 | 0.09 |
| Transverse temporal (L) | -0.04 | -0.05 | 0.01 | 0.04 |
| Caudal middle frontal (R) | 0.03 | 0.09 | 0.06 | 0.06 |
| Cuneus (R) | 0.09 | 0.09 | 0.06 | 0.1 |
| Fusiform (R) | -0.03 | 0.06 | 0.02 | 0.1 |
| Inferior parietal (R) | 0.01 | 0.07 | 0.03 | 0.09 |
| Lateral occipital (R) | 0.13 | 0.13 | 0.11 | 0.17 |
| Lateral orbitofrontal (R) | -0.06 | 0.07 | 0 | 0.05 |
| Medial orbitofrontal (R) | -0.04 | -0.01 | -0.03 | 0.04 |
| Middle temporal (R) | -0.16 | -0.05 | -0.1 | -0.01 |
| Paracentral (R) | 0.02 | 0.08 | 0.04 | 0.12 |
| Parahippocampal (R) | -0.07 | -0.01 | -0.01 | 0.02 |
| Posterior cingulate (R) | -0.04 | 0.02 | -0.01 | 0.08 |
| Precuneus (R) | 0.1 | 0.18 | 0.09 | 0.19 |
| Rostral middle frontal (R) | -0.03 | 0.01 | -0.01 | 0.03 |
| Superior frontal (R) | -0.09 | 0 | -0.04 | 0.06 |
| Superior parietal (R) | 0.06 | 0.1 | 0.06 | 0.14 |
| Superior temporal (R) | -0.08 | 0.01 | -0.01 | 0.05 |
| Supramarginal (R) | 0 | 0.12 | 0.02 | 0.1 |
| Transverse temporal (R) | -0.04 | 0.01 | -0.01 | 0.07 |

**
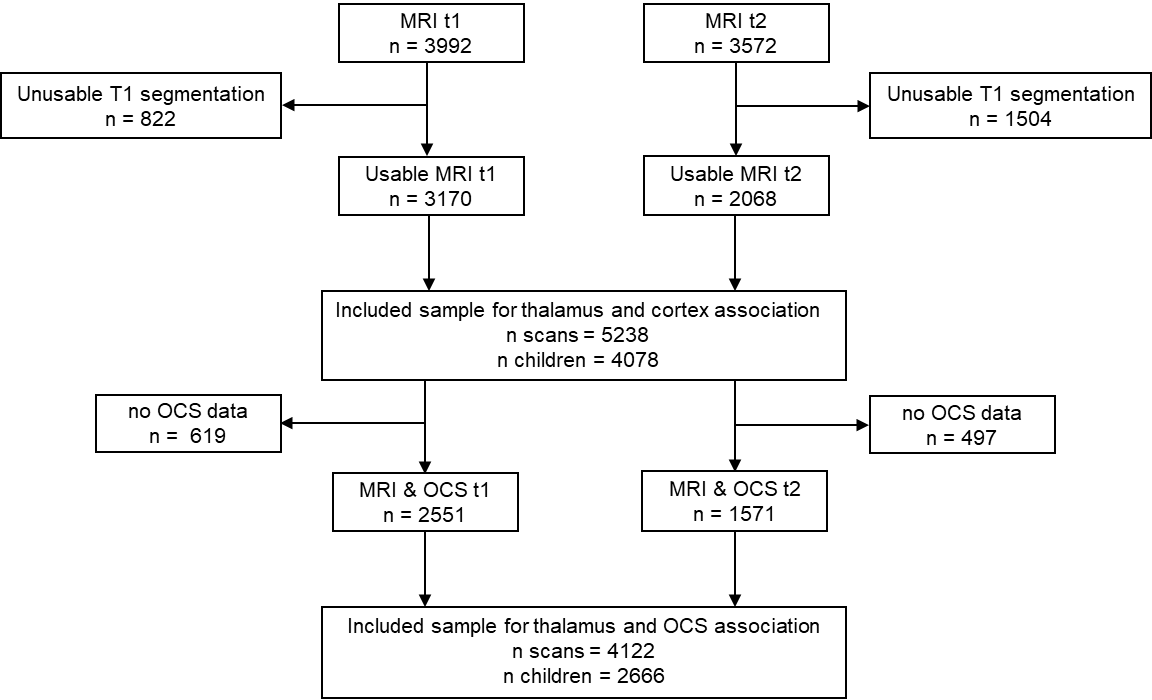
Figure S1**

Figure S1: Flowchart depicting the initial sample size, exclusion criteria and final sample size.

**Figure S2**


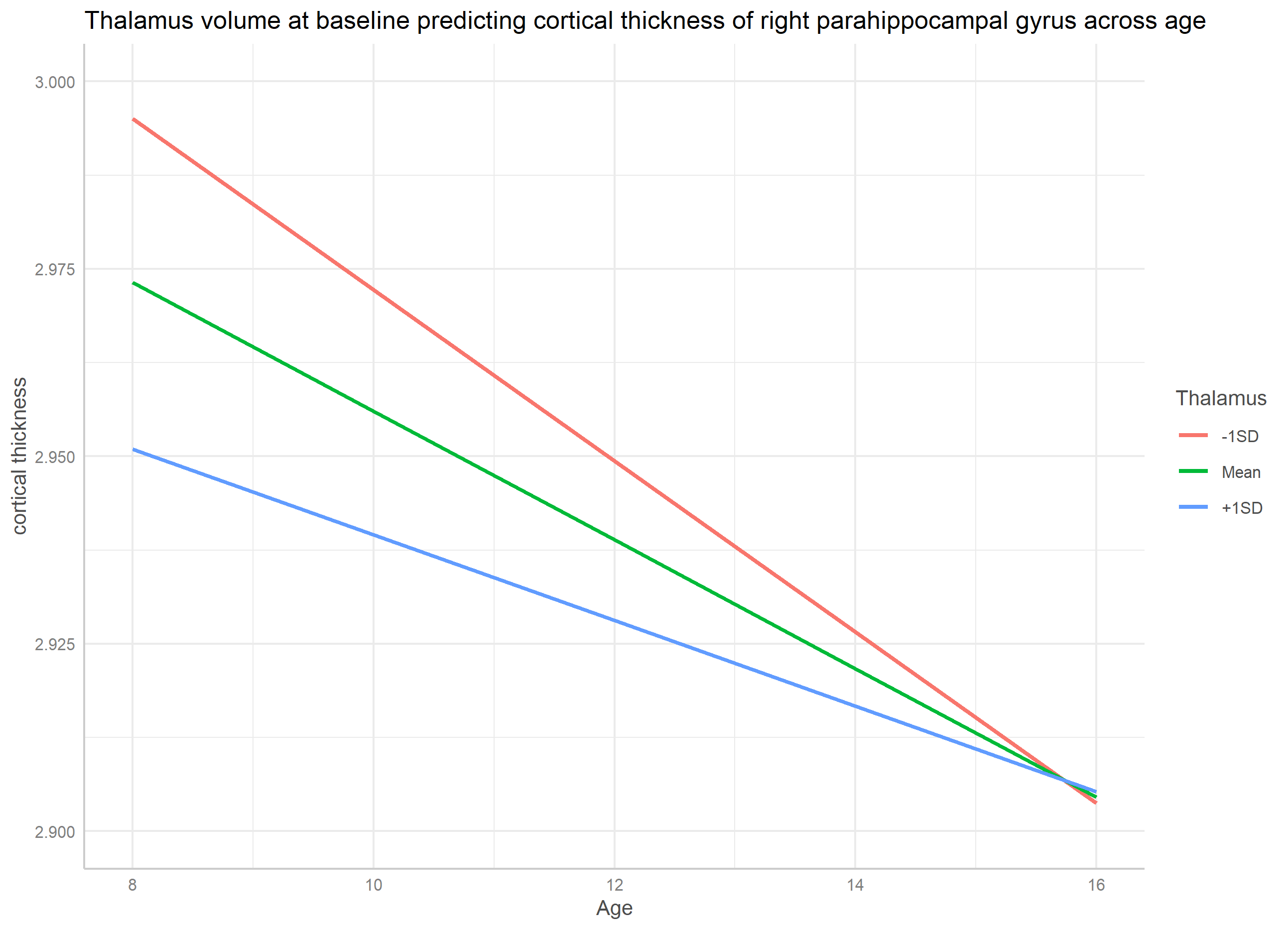


Figure S2: Visualization of the predicted model estimates derived from linear mixed-effects models for cortical thickness of the parahippocampal gyrus. The y-axis represents the cortical metric based on model estimates, and lines represent different levels of mean thalamic volume.

**Figure S3**


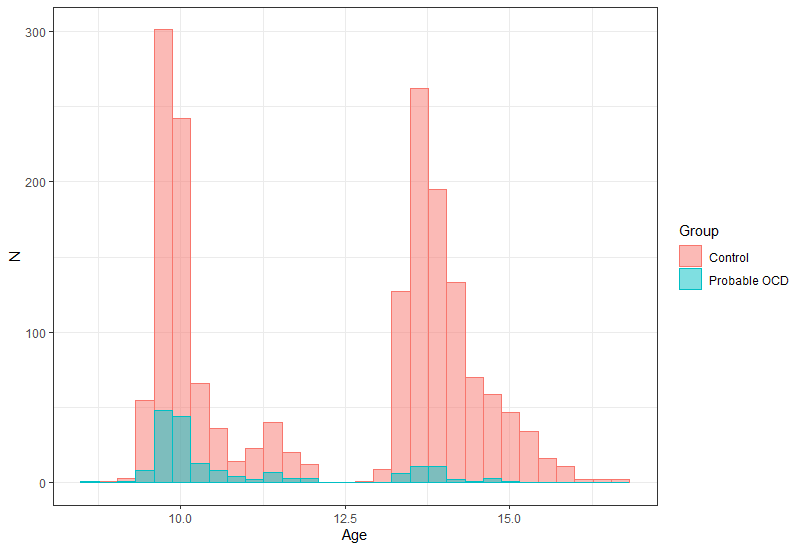


Figure S3: Age distribution in expanded cross-sectional dataset spanning both time points. Age follows a bimodal distribution and the number of probable OCD participants at higher ages is lower than at the first ages.
